# Dual‐Functionalized Extracellular Vesicles Promote Brain Repair and Remodeling Following Ischemic Stroke in Mice

**DOI:** 10.1111/cns.70597

**Published:** 2025-09-15

**Authors:** Victoria Shi, Shengju Wu, Qianyuan Lian, Rubing Shi, Ze Liu, Tongtong Xu, Shiyu Deng, Xinfa Shao, Anja Beckmann, Wanlu Li, Yaohui Tang, Carola Meier, Guo‐Yuan Yang, Zhijun Zhang

**Affiliations:** ^1^ Shanghai Jiao Tong University Affiliated Sixth People's Hospital, Med‐X Research Institute and School of Biomedical Engineering Shanghai Jiao Tong University Shanghai China; ^2^ Department of Anatomy and Cell Biology, Medical Faculty Saarland University Homburg/Saar Germany

**Keywords:** bioengineering, drug delivery, extracellular vesicles, ischemic stroke, RGD peptide, stem cell

## Abstract

**Background:**

Ischemic stroke remains a leading cause of long‐term disability and mortality worldwide, with few effective treatment options. A key challenge in recovery is the brain's limited capacity to regenerate neurovascular structures after injury. To address this, we developed a dual‐functionalized extracellular vesicle (EV) platform designed to enhance both targeting specificity and therapeutic efficacy for post‐stroke repair.

**Methods:**

Neural stem cell‐derived EVs were bioengineered via bio‐click chemistry to display RGD peptides, enabling selective binding to integrin αVβ3, which is upregulated on activated endothelial cells in ischemic regions. EVs were concurrently loaded with vascular endothelial growth factor (VEGF), a pro‐angiogenic and neurogenic cytokine that also enhances αVβ3 expression—thus creating a synergistic positive feedback mechanism to amplify targeting and tissue repair.

**Results:**

Engineered EVs retained normal morphology and showed a 5.2‐fold increase in endothelial uptake compared to naïve EVs (*p* < 0.01). In vitro, they significantly enhanced endothelial cell migration by 2.1‐fold (*p* < 0.05). In a mouse model of transient middle cerebral artery occlusion (tMCAO), intravenously delivered dual‐functionalized EVs preferentially accumulated in the ischemic hemisphere, reduced infarct volume by 52.4%, and improved motor coordination (rotarod latency) by 71.8% compared to PBS‐treated controls (*p* < 0.05). Immunostaining revealed enhanced CD31+ microvessel density and increased Nestin+ neural stem and progenitor cell presence, indicating promotion of both angiogenesis and neurogenesis.

**Conclusion:**

This study presents a dual‐functionalized EV system that combines targeted delivery with therapeutic reinforcement through VEGF loading, offering a potent and synergistic approach for ischemic stroke repair. These findings support further translational development of engineered EVs for neurovascular regeneration.

## Graphical abstract

RGD‐VEGF‐EVs reduce infarct volume and promote neurobehavioral outcomes by angiogenesis in a mouse model of ischemic stroke.

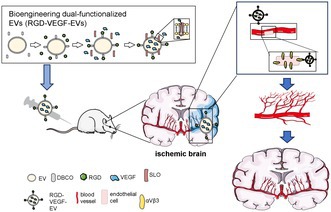

**Abbreviations**
BBB: blood–brain barrier
bFGF: basic fibroblast growth factor
CBF: cerebral blood flow
CCK‐8: Cell Counting Kit‐8
DBCO: dibenzylcyclootyne
dFBS: EV‐depleted FBS
DIR: [1,1‐dioctadecyl‐3,3,3,3‐tetramethylindotricarbocyanine iodide]
ECA: external carotid artery
EGF: epidermal growth factor
EVs: extracellular vesicles
FBS: fetal bovine serum
HUVECs: human umbilical cord endothelial cells
ICA: internal carotid artery
IF: immunofluorescence staining
IVIS: in vivo imaging system
LDF: Laser Doppler flowmetry
MCA: middle cerebral artery
mNSS: Modified Neurological Severity Score
NIRF: near‐infrared fluorescence
NSCs: neural stem cells
NTA: nanoparticle tracking analysis
PFA: paraformaldehyde
RGD: Arg‐Gly‐Asp
ROI: region of interest
RT: room temperature
SD: standard deviation
SLO: Streptolysin‐O
TCEP: tris (2‐carboxyethyl) phosphine
TEM: transmission electron microscope
tMCAO: transient middle cerebral artery occlusion
VEGF: vascular endothelial growth factor

## 1 Introduction

Stroke remains the second leading cause of death and a leading cause of adult disability worldwide [1]. Ischemic stroke, caused by the occlusion of cerebral arteries, accounts for approximately 65% of all stroke cases [1]. Despite its prevalence, effective treatment options remain limited.

Currently, the only FDA‐approved pharmacological treatment for ischemic stroke is intravenous thrombolysis with recombinant tissue plasminogen activator (rtPA). However, its clinical application is severely restricted by a narrow 4.5‐hour treatment window, a high risk of hemorrhagic complications, and strict eligibility criteria, ultimately rendering only about 5% of all stroke patients eligible for treatment [2, 3]. This therapeutic gap underscores the urgent need for alternative strategies that extend the treatment window and promote post‐stroke neurovascular regeneration.

Extracellular vesicles (EVs) have emerged as promising candidates for ischemic stroke treatment [4]. EVs are nanosized vesicles (30–200 nm), enclosed by a lipid bilayer. They are secreted by cells to transport cargo from the donor to the recipient cell for cell–cell communication. EVs are advantageous to stem cell therapy as clogging of microvessels or unpredictable cell proliferation is avoided [5, 6, 7]. EVs exhibit low immunogenicity, minimal toxicity, and the ability to cross the blood–brain barrier (BBB)—a significant obstacle in the treatment of neurological diseases [8, 9, 10, 11].

EVs derived from neural stem cells (NSC‐EVs) can enhance neuronal survival, reduce inflammation, modulate the immune microenvironment, promote angiogenesis, and inhibit apoptosis of brain cells [11, 12, 13]. Despite these benefits, one of the key limitations of naive EVs is their poor ability to accumulate specifically in ischemic brain regions, which greatly reduces their therapeutic efficiency [8, 12].

To overcome this limitation, researchers turned to surface modification techniques to improve EV targeting capacity. One effective strategy is copper‐free bio‐click‐chemistry, which enables the covalent conjugation of a site‐specific targeting peptide on EV surface through triazole formation, without affecting EV integrity or biological activity [14]. Among various ligands, the Arg‐Gly‐Asp (RGD‐) peptide is of particular interest due to its high binding affinity to integrin αVβ3. αVβ3 is a receptor selectively upregulated on activated endothelial cells in ischemic tissue and plays a central role in angiogenesis [15]. Thus, functionalizing EVs with the RGD peptide enhances their accumulation in ischemic brain regions and increases their therapeutic impact [15, 16, 17, 18].

In addition to improving EV targeting, enhancing the therapeutic payload of EVs is critical to maximizing their regenerative potential [12]. Among candidate molecules, the vascular endothelial growth factor (VEGF) is a well‐characterized pro‐angiogenic and neuroprotective cytokine that not only promotes vascular remodeling and neurogenesis but also upregulates integrin αVβ3 expression, potentially reinforcing RGD‐mediated targeting via a positive feedback mechanism [15, 19, 20]. However, conventional loading techniques such as sonication [21] or incubation [22] show low loading efficiencies of less than 30% [21, 22]. To address this issue, researchers have turned to Streptolysin‐O (SLO), a cytolysin that binds to cholesterol‐rich membranes. There, it oligomerizes and forms transient pores of less than 30 nm in diameter, creating reversible permeabilization for drugs. These pores allow for efficient loading of drugs into EVs, significantly improving encapsulation compared to passive methods [23, 24].

In this study, we propose a dual‐functionalized, bioengineered NSC‐EV‐based nanoplatform for the treatment of ischemic stroke. Our approach consists of two key strategies: (1) Surface functionalization of NSC‐EVs with the RGD‐peptide using copper‐free bio‐click chemistry to improve targeted delivery to the ischemic brain; (2) SLO‐mediated loading of VEGF into these RGD‐functionalized EVs to boost angiogenic and neuroregenerative remodeling, neuroprotection, as well as to further upregulate αVβ3 expression in endothelial cells [15, 19, 20].

We hypothesize that the combination of targeted delivery, via RGD surface functionalization, with enhanced therapeutic cargo loading, via SLO‐mediated VEGF encapsulation, will synergistically improve accumulation of NSC‐EVs in ischemic brain regions and amplify their neuroregenerative effects, offering a novel and clinically translatable therapeutic strategy for ischemic stroke.

## 2 Materials and Methods

### 2.1 Primary NSC Isolation and Culture

NSCs were isolated from the cortices of embryonic day 15 (E15) C57/Bl6J mice. The cortices were digested with 0.125% w/w Trypsin–EDTA at 37°C. Digestion was stopped with 5% FBS, and a single‐cell suspension was created by mechanical disruption. After centrifugation (4 min, 125 ×*g*), the cells were resuspended in the serum‐free DMEM/F12 (1:1) medium with 1% B‐27, 20 ng/mL basic fibroblast growth factor (bFGF), 20 ng/mL epidermal growth factor (EGF), and 1% penicillin G (100 units/mL) and streptomycin (100 μg/mL). The cells were kept at 37°C in a humidified 5% CO_2_ incubator.

### 2.2 EV Isolation, Characterization, and Labelling

EVs were isolated from the conditioned medium of NSCs from P0 to P5 via ultracentrifugation as previously described [25]. The centrifugation steps are as follows: 300 ×*g* for 10 min, 2000 ×*g* for 10 min, and 10,000 ×*g* for 35 min. Afterward, the supernatant was ultracentrifuged (Optima XPN‐100, Beckman Coulter, USA) at 100,000 ×*g* for 70 min. The supernatant was discarded, EVs were washed in PBS, and ultracentrifuged again. EVs were resuspended and kept in PBS at 4°C until use.

EV‐specific proteins TSG101 (ab125011, abcam, USA) and CD9 (ET1601‐9, Huabio, China) were detected in a Western blot analysis with GM130 (sc‐55591, Santa Cruz, USA) as the negative control. EV morphology was shown with a transmission electron microscope (TEM, Tecnai G2 Spirit BioTWIN, Thermo Scientific, USA). EV size was detected via nanoparticle tracking analysis (NTA, Zetaview x30, Particle Metrix, Germany).

For PKH26 labelling, EVs were incubated with 2 μM PKH26 (HY‐D1451, MCE, USA) at room temperature (RT) for 4 min. Excess labelling was stopped with EV‐depleted FBS (dFBS). For DIR labelling, EVs were incubated with 4 μM DIR (MB12482, MeilunBio, China) at RT for 8 min. Afterward, EVs were washed with PBS and ultracentrifuged twice at 100,000 ×*g* for 70 min each.

### 2.3 EV Engineering and Characterization

The RGD‐peptide was linked to the EV surface via copper‐free bio‐click chemistry as previously described, with minor adjustments [26]. First, reactive dibenzylcyclooctyne (DBCO, 764019, sigma‐aldrich, USA) was conjugated on the surface of EVs. Therefore, 0.75 mg/mL EVs reacted with 2 mM DBCO‐PEG_4_‐NHS at RT for 4 h. Afterward, unbound DBCO was eliminated by centrifugation at 12,000 ×*g* three times for 10 min each using ultrafiltration tubes (3.5 kDa, 88512, Thermo Scientific, USA). Cyclo(RGDfK(N_3_))‐peptide (Apeptide, China) was incubated in a 1.5‐fold concentration to DBCO on a shaker at 4°C overnight. The undecorated peptide was eliminated using ultrafiltration as described before.

SLO was used for EV loading as previously described [23]. First, 2 U/mL SLO (HY‐135416, MCE) was activated with 5 mM tris (2‐carboxyethyl) phosphine (TCEP, T2556, Thermo Fisher Scientific) at 37°C for 1 h. For loading, 0.6 mg/mL EVs were incubated with 0.2 U/mL activated SLO and 1 μM VEGF at 37°C for 2 h. Unloaded VEGF was removed via ultrafiltration. Successful bioengineering of EVs was verified using immunofluorescence staining (IF), measurement of zeta potential, and Western blot. Loading efficiency was determined with BCA and the drug release profile with ELISA. Bioengineered EVs were freshly prepared for each experiment.

### 2.4 Cytotoxicity of Bioengineered EVs In Vitro

Cell viability was determined using the Cell Counting Kit‐8 (CCK‐8, MA0218, MeilunBio). 1X10^4^ Human umbilical vein endothelial cells (HUVECs) were seeded per well in a 24‐well plate one day prior to the experiment. Cells cultured in DMEM with 10% dFBS were stimulated with bioengineered EVs in concentrations up to 200 μg/mL for 24 h. Afterward, the cell culture medium was replaced with 10% CCK‐8 solution in DMEM, followed by incubation at 37°C for 1 h. The absorbance at 450 nm was measured using a microplate reader (Synergy H1, Agilent BioTek, China) as described previously [27].

### 2.5 Uptake of Bioengineered EVs In Vitro

8X10^3^ HUVECs were seeded onto 12 mm diameter glass coverslips one day prior to the experiment. Cells were incubated with 20 μg/mL PKH26‐labeled EVs for 3 h. To block αVβ3‐binding sites, HUVECs were incubated with 15 μg/mL RGD prior to EV stimulation for 30 min. Cells were fixed with 4% paraformaldehyde (PFA) in PBS at RT for 10 min and mounted with the mounting medium containing DAPI. Images of 4 random positions per coverslip were taken, and their fluorescence intensity was analyzed using Image*J* 1.54f (NIH, USA) [28].

To verify cellular uptake of EVs, 3D images were taken with a confocal microscope (IX70 Fluoview, Olympus, Japan).

### 2.6 Cell Migration Assays

#### 2.6.1 Scratch Assay

1X10^5^ HUVECs were seeded per well of a 24 well plate one day prior to the experiment. A scratch was made through the cell monolayer using a 200 μL pipette tip. Cells were washed twice in PBS and incubated with 20 μg/mL bioengineered EVs in DMEM with 10% dFBS. Phase contrast images were taken at the start of migration and 9 h later at marked positions. Cell migration at 5 positions across the scratch was measured and averaged.

#### 2.6.2 Transwell Migration Assay

1X10^4^ HUVECs were seeded per transwell (Corning 3422, USA). Transwells were then placed in 500 μL DMEM with 10% dFBS and 20 μg/mL bioengineered EVs. After 16 h, non‐migratory cells on the upper membrane were cleared away with a cotton swab. Migrated cells on the lower membrane were fixed in 4% PFA at RT for 10 min and stained with 10 μg/mL DAPI (MA0127, MeilunBio). Five random fields were imaged with a fluorescent microscope to quantify migration as described previously [26].

### 2.7 Transient Middle Cerebral Artery Occlusion (tMCAO)

Animal procedures were performed in accordance with ARRIVE guidelines and the guidelines of the Institutional Animal Care and Use Committee (IACUC) of Shanghai Jiao Tong University, Shanghai, China (animal ethics protocol number 2023031; approval date: December 08, 2023). Mice were anesthetized with an air mixture of 1.5 to 2% isoflurane and 30%/70% oxygen/nitrous oxide. Transient middle cerebral artery occlusion (tMCAO) was performed as previously described [29]. Briefly, the common carotid artery, internal carotid artery (ICA), and external carotid artery (ECA) were isolated. A lysine‐coated nylon filament (A4‐162250, Beijing Cinotech, China) was inserted from the ECA into the ICA to the origin of the middle cerebral artery (MCA). Surface cerebral blood flow (CBF) was measured with laser Doppler flowmetry (LDF, Moor Instruments, UK). Occlusion was regarded as successful when CBF decreased to 10% to 20% of baseline CBF. The suture was withdrawn after 90 min. Surgery was regarded as successful when CBF recovered to 70% of baseline CBF.

### 2.8 Animal Experiment Design

Adult 87 male eight‐week‐old C57/Bl6 mice (*n* = 8, 22‐25 g) were randomly divided into six groups: (1) Sham, (2) PBS, (3) naïve EVs, (4) RGD‐EVs, (5) VEGF‐EVs, (6) RGD‐VEGF‐EVs. Sham mice underwent no surgery. Mice in EV groups were injected with 100 μg EVs in 100 μL PBS through the tail vein every other day from D7 to D14 after tMCAO. Mice in the PBS group were injected with 100 μL PBS. All mice underwent neurobehavior testing. Animals were sacrificed 15 days following tMCAO. Brains were perfused with PBS, extracted, and frozen in pre‐cooled isopentane. The mortality rate of the tMCAO model in this study is 25%.

### 2.9 Neurobehavioral Tests

Neurobehavioral tests were assessed 1 day prior, 1 day after injury, on D7, and D14 after tMCAO by a researcher blinded to animal treatment. A Modified Neurological Severity Score (mNSS) testing for motor, balance, and reflex functions was determined on a scale from 0 (normal) to 12 (severe injury) [30].

Balance and motor coordination were further tested with the rotarod and balance beam tests. For the rotarod test, mice were first allowed to adapt on the rod for 1 min. Afterward, the rod was accelerated to 40 rpm, and mice ran for a maximum of 5 min. The time mice spent on the rod was recorded. Mice received 3 days of training prior to the first testing with 3 training rounds each. On the first day of training, the rod was accelerated to 20, 25, and 30 rpm; on the second day to 25, 30, and 35 rpm; and on the third day to 30, 35, and 40 rpm, for 5 min each. For the balance beam test, a narrow walking beam of 6 mm width was placed 40 cm above the ground. The time required to traverse a 50 cm length of the beam was recorded.

Muscle strength was assessed via the hanging wire test as previously described [31]. A horizontal wire 1.6 mm in diameter, 50 cm in length, was hung 30 cm above the ground. Mice grabbed the wire with their fore limbs. The cut‐off time for this test was 3 min. Average hanging time was analyzed.

### 2.10 Infarct Volume Assessment

Coronal cryosections, 10 μm thickness, 100 μm apart, were stained with Nissl staining solution (MA0129, MeilunBio) at RT for 12 min. Slices were washed under running water, air‐dried, and scanned. The area of the ipsilateral and contralateral hemispheres was measured using Image*J* 1.54 f. Infarct volume was calculated by subtracting the area of the ipsilateral hemisphere from the contralateral hemisphere and multiplying it by the interval thickness between two slices according to the following formula:

$$v=\frac{h}{3}*\sum_{1}^{n}\left[\left(s_{n}+\sqrt{s_{n}*s_{n+1}}+s_{n+1}\right)\right]$$

with *h* as the interval between two slices and *s* as the hemisphere area of each brain slice. Data were analyzed using GraphPad Prism 9 (GraphPad Software, USA). A one‐way ANOVA was used to compare atrophy volume across all treatment groups of mice, followed by Tukey's post hoc test for pairwise comparisons between experimental groups of mice treated with PBS, naïve EVs, RGD‐EVs, VEGF‐EVs, and RGD‐VEGF‐EVs after ischemic stroke. Statistical significance was set at *p* < 0.05. The sample size was five mice per group.

### 2.11 Biodistribution of Bioengineered EVs In Vivo

DIR‐labeled EVs (100 μg) were injected into the mouse tail vein 7 days after tMCAO. Mice were sacrificed 6 h after injection, perfused with PBS, and organs were harvested and fixed in 4% PFA. Near‐infrared fluorescence imaging was performed with an in vivo imaging system (IVIS) instrument (PerkinElmer, USA) to determine the biodistribution of EVs. Regions of interest (ROI) were analyzed using Living Image software (Vers 4.7.2, Perkin Elmer).

### 2.12 Immunofluorescence Staining

Cells or cryosections were fixed in 4% PFA at RT for 10 min, permeabilized with 0.3% TritonX‐100 in PBS for 25 min, and blocked with 5% BSA at RT for 1 h. The primary antibody was incubated overnight in QuickBlock primary antibody diluent (P0262, Beyotime, China). The secondary antibody was diluted in PBS at 37°C in the dark for 1 h. Primary antibodies used are CD31 (AF3628, R&D, USA), NeuN (PRB039, Oasis, China), CD11b (101201, Biolegend, USA), GFAP (PGP055, Oasis), nestin (MAB353, Millipore, USA), and Ki‐67 (14–5698‐82, Invitrogen, USA). Secondary antibodies used are anti‐rabbit 647 (A31573, Invitrogen), anti‐goat 488 (A11055, Invitrogen), anti‐rat 488 (A21208, Invitrogen), anti‐mouse 488 (A21202, Invitrogen), and anti‐guinea pig 488 (G‐GP488, Oasis).

### 2.13 Statistical Analysis

Data are presented as mean ± standard error of mean (SEM). Normal distribution was tested with the Kolmogorov–Smirnov test using GraphPad Prism 9. Further analyses performed are one‐way or two‐way ANOVA with Tukey's multiple comparisons test. A *p*‐value below 0.05 was considered statistically significant.

## 3 Results

### 3.1 Preparation and Characterization of Bioengineered EVs

**FIGURE 1:**
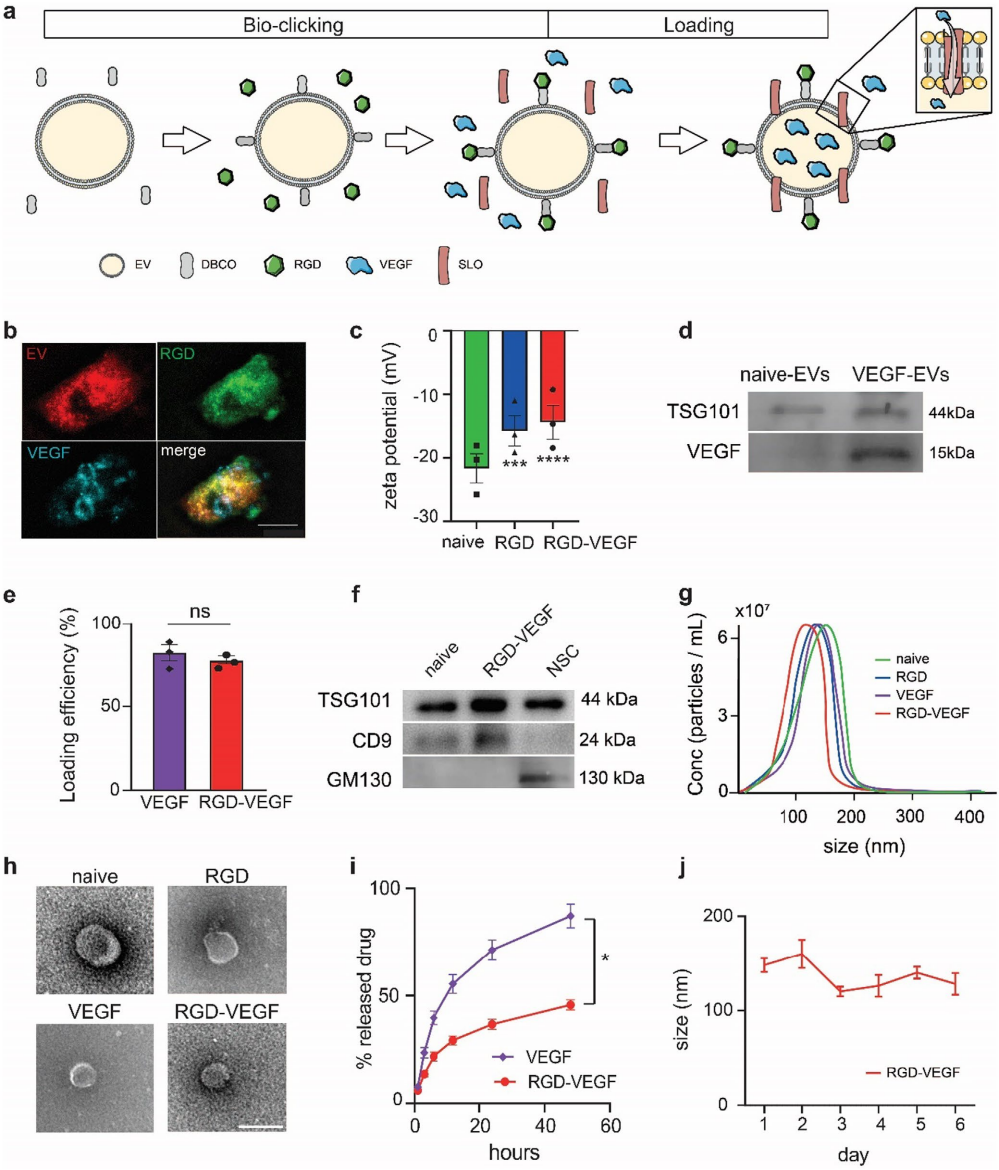
Characterization of bioengineered EVs. (a) In the first engineering step, RGD is conjugated to the EV surface via bio‐click chemistry. Therefore, DBCO is first conjugated to the EV surface. Next, the RGD peptide binds to DBCO on the EV surface. In the second bioengineering step, RGD‐functionalized EVs are loaded with VEGF using Streptolysin‐O (SLO). (b) Representative fluorescence image with single channel images of EVs labeled with PKH26 (red), FITC‐RGD peptide (green), and VEGF labeled with Cy5.5 (cyan) as well as merged image. Scale bar 20 μm. (c) Measurement of zeta‐potential of naïve, RGD‐ and RGD‐VEGF‐EVs. Statistical significances: ***p < 0.001; ****p < 0.0001 vs. naïve EVs. (d) Western blot analysis of naïve EVs and VEGF‐EV for VEGF and TSG101 as control. (e) Quantification of VEGF‐loading efficiency of VEGF‐EVs and RGD‐VEGF‐EVs measured with BCA. ns = not significant. (f) Western blot analysis of naïve‐EVs, RGD‐VEGF‐EVs, and NCS cell lysate for EV‐characteristic proteins TSG101 and CD9, as well as GM130 as a negative control. (g) Nanoparticle tracking analysis (NTA) showing size distribution and particle concentration of naïve EVs, RGD‐EVs, VEGF‐EVs, and RGD‐VEGF‐EVs. (h) Transmission electron microscopy (TEM) images of naïve EVs, RGD‐EVs, VEGF‐EVs, and RGD‐VEGF‐EVs reveal EV‐typical cup‐shape throughout the bioengineering procedure. Scale bar 100 nm. (i) VEGF drug release profile of VEGF‐EVs and RGD‐VEGF EVs measured with ELISA assay. Statistical significances: *p < 0.05 vs. Control. (j) Mean particle size of RGD‐VEGF‐EVs monitored over 6 consecutive days after engineering via NTA. *N* = 3 per experiment.

For bio‐click chemistry, DBCO‐PEG_4_‐NHS is conjugated covalently with NSC‐EV surface proteins. Afterward, the RGD‐N_3_ peptide was introduced, which formed stable triazole linkages with DBCO on the EV surface. Naïve or RGD‐EVs were loaded with VEGF using the pore‐forming bacterial toxin SLO (Figure 1a). Fluorescence labeling, zeta‐potential measurement, and Western blot confirm successful bioengineering. The overlap of FIT‐RGD, Cy5.5‐VEGF, and PKH26‐EVs visually confirms successful bioengineering (Figure 1b) as well as an increase in electrical potential after bio‐clicking the positively charged RGD‐peptide on the negatively charged EV surface (Figure 1c). Western blot shows successful loading of EVs with VEGF (Figure 1d). The loading efficiency for both VEGF‐EVs and RGD‐VEGF‐EVs is over 70% (Figure 1e).

Naïve as well as bioengineered EVs are both positive for EV‐characteristic proteins TSG101 and CD9 and negative for the Golgi protein GM130 (Figure 1f). The shape and size of bioengineered EVs were similar among groups (Figure 1g). TEM analysis confirms the EV‐typical cup shape for all bioengineered EVs (Figure 1h). Interestingly, VEGF‐EVs release more than 85% of initially loaded VEGF after 48 h, while RGD‐VEGF‐EVs release less than 50% (Figure 1i). RGD‐VEGF‐EVs stored in PBS at 4°C are stable in size up to 6 days after engineering (Figure 1j).

### 3.2 Cytocompatibility, Uptake, and Migratory Effects of Bioengineered EVs In Vitro

**FIGURE 2:**
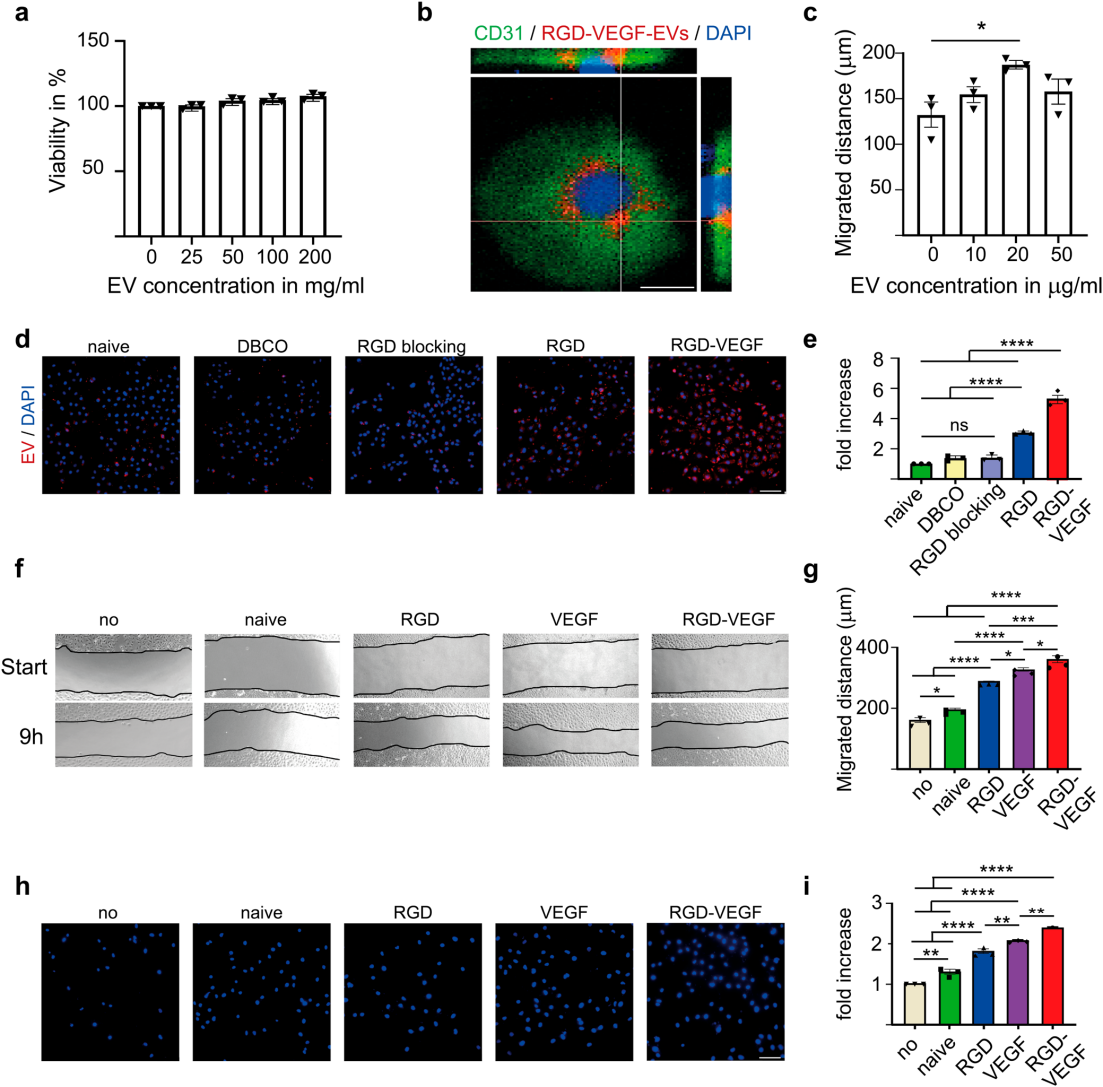
In vitro cytocompatibility, cellular uptake, and migratory effects of bioengineered EVs on endothelial cells. (a) HUVEC cell viability after stimulation with RGD‐VEGF‐EVs at various concentrations (0, 25, 50, 100, and 200 μg/mL) for 24 h, assessed using CCK‐8. (b) Representative 3D confocal image showing internalization of PKH26‐labeled RGD‐VEGF‐EVs by HUVECs. Endothelial cell marker CD31 in green, EVs in red, nuclei in blue (DAPI). Scale bar 20 μm. (c) Quantification of migration of HUVECs treated with NSC‐EVs at various concentrations (0, 10, 20, and 50 μg/mL) for 9 h in a scratch assay, expressed in absolute distance. (d) Representative images of bioengineered EV uptake by HUVECs after 3 h incubation with EVs in red (PKH26), nuclei in blue (DAPI). Scale bar 100 μm. (e) Quantification of EV uptake shown in (d), normalized to naïve EVs. (f) Representative brightfield images from the scratch migration assay of HUVECs treated with 20 μg/mL bioengineered EVs for 9 h. (g) Quantification of migration shown in (f), expressed as absolute distance. (h) Representative images of DAPI signals of migrated HUVECs after 16 h stimulation with 20 μg/mL bioengineered EVs in a transwell migration assay. Scale bar 100 μm. (i) Quantification of migration of HUECs shown in (h), normalized to migration without EV stimulation. *N* = 3 per experiment. Statistical significances: ns= not significant *p < 0.05; **p < 0.01; ***p < 0.001; ****p < 0.0001 vs. Control.

HUVEC viability does not change after EV treatment with RGD‐VEGF‐EVs for 24 h (Figure 2a). 3D confocal imaging confirms internalization of EVs in vitro (Figure 2b). We determined a suitable dose of 20 μg/mL EVs for further in vitro experiments, as this dose increases HUVEC migration after 9 h (Figure 2c). We then analyzed the uptake of PKH26‐stained bioengineered EVs in HUVECs after 3 h (Figure 2d). To confirm that RGD‐αVβ3 targeting causes an increase in EV uptake, two control groups, DBCO and RGD‐blocking, were added. In the DBCO group, only DBCO was linked to the EV surface, without the RGD peptide. In the RGD‐blocking group, HUVECs were first incubated with RGD to block αVβ3 binding sites before RGD‐EV incubation. Fluorescence intensity was normalized to naïve EVs. Uptake of DBCO‐EVs and EVs in the RGD‐blocking condition is not increased. The uptake of RGD‐EVs is 3‐fold, and of RGD‐VEGF‐EVs is 5.2‐fold (Figure 2e).

For the scratch migration assay, the migrated distance was analyzed after stimulation with bioengineered EVs for 9 h (Figure 2f). RGD‐VEGF‐EVs increase cell migration compared to stimulation with naïve or single‐functionalized EVs (Figure 2g).

In the transwell migration assay, migrated HUVECs were analyzed after stimulation with bioengineered EVs for 16 h (Figure 2h). HUVEC migration was increased 2.1‐fold compared to stimulation without EVs. Similar to the results from the scratch test, RGD‐VEGF‐EVs significantly increase cell migration compared to stimulation with naïve or single‐functionalized EVs (Figure 2i).

### 3.3 Bio‐Distribution of Bioengineered EVs In Vivo

**FIGURE 3:**
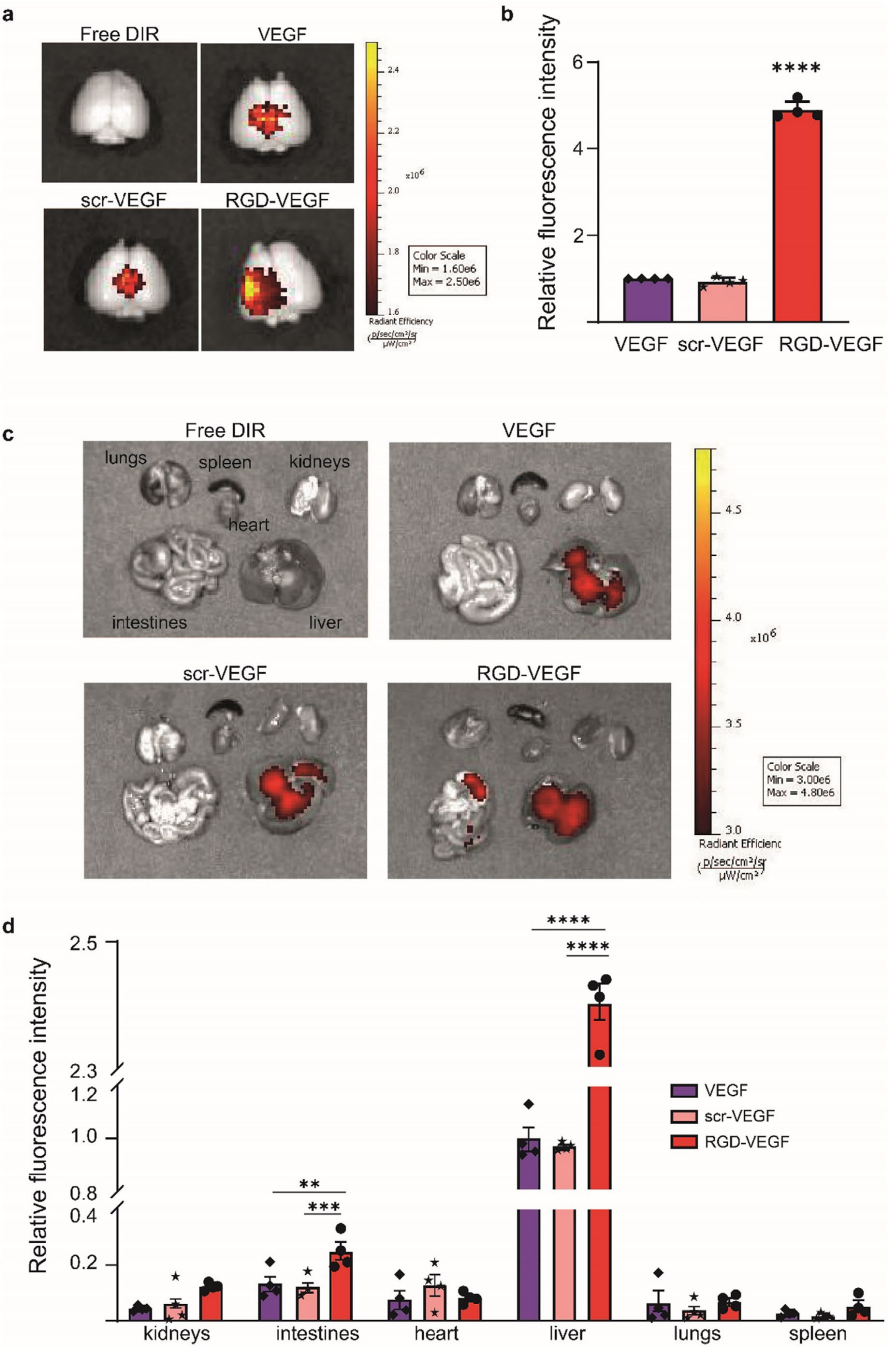
Targeting abilities of bioengineered EVs to the ischemic brain and their biodistribution in major visceral organs determined by IVIS. (a) White light images with overlain radiant efficiency of mouse brains with DIR‐labeled VEGF‐EVs, scrambled RGD‐peptide‐conjugated VEGF‐EVs (scr‐VEGF‐EVs), and RGD‐VEGF‐EVs. (b) Quantification of radiant efficiency shown in (a) normalized to the radiant efficiency of VEGF‐EVs. Statistical significance: ****p < 0.0001 compared to VEGF‐EVs. (c) Organ‐level biodistribution of DIR‐labeled VEGF‐EVs, scr‐VEGF‐EVs, and RGD‐VEGF‐EVs in major visceral organs. (d) Quantification of the radiant efficiency of (c). *N* = 4 per group. Statistical significances: *p < 0.05; **p < 0.01; ***p < 0.001; ****p < 0.0001 vs. Control.

We determine bio‐distribution and targeting abilities of Cy5.5‐labeled EVs using IVIS. Compared to VEGF‐EVs, the radiant efficiency of RGD‐VEGF‐EVs is 5‐fold higher in the brain (Figure 3a,b). The majority of RGD‐VEGF‐EVs accumulate in the liver and intestines (Figure 3c,d). scr‐VEGF‐EVs and VEGF‐EVs are taken up at comparable rates.

### 3.4 Cellular Uptake of Bioengineered EVs in Mice

**FIGURE 4:**
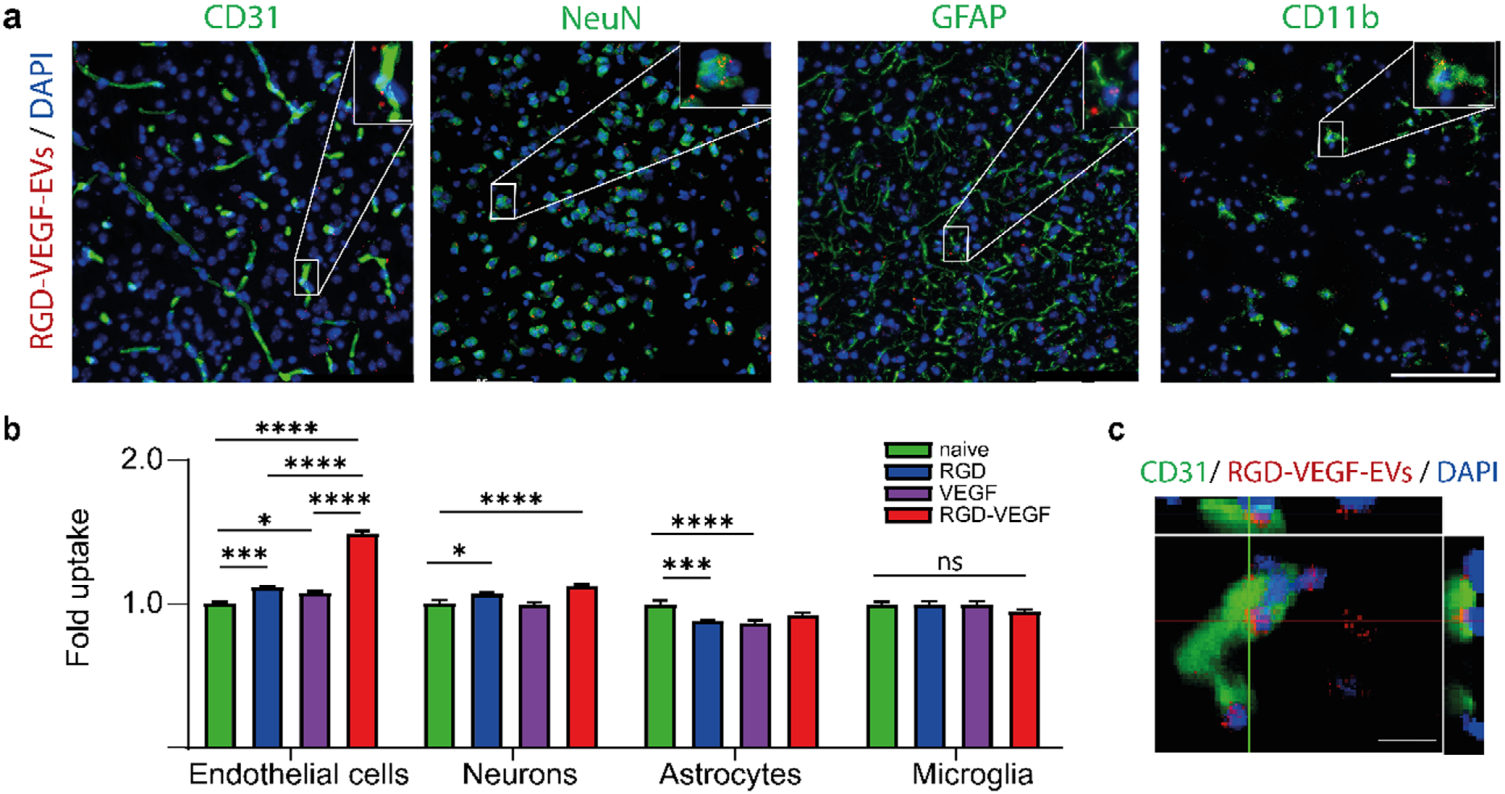
Cellular uptake of bioengineered EVs in vivo. (a) Representative images showing uptake of RGD‐VEGF‐EVs (PKH26, red) by endothelial cells (CD31, green), neurons (NeuN, green), astrocytes (GFAP, green), and microglia (CD11b, green), including corresponding magnified views with (b) bar graph showing quantification of EV uptake normalized to uptake of naive EVs. Scale bars 100 μm, scale bars 10 μm in magnification. *N* = 6–8 per group. Statistical significances: *p < 0.05; ***p < 0.001; ****p < 0.0001 vs. Control. (c) Representative 3D confocal image showing internalization of PKH26‐labeled RGD‐VEGF‐EVs by endothelial cells. Endothelial cell marker CD31 in green, nuclei in blue (DAPI), scale bar 20 μm. *N* = 6–8. Statistical significances: *p < 0.05; ***p < 0.001; ****p < 0.0001 vs. Control.

Next, we investigated in vivo cellular uptake of bioengineered EVs. Immunostaining was performed on cryosections to stain for endothelial cells (CD31), astrocytes (GFAP), neurons (NeuN), and microglia (CD11b). All four cell types internalize EVs (Figure 4a). Compared to the uptake of naïve EVs, endothelial cells show a 1.5‐fold uptake of RGD‐VEGF‐EVs. Neurons show a 1.1‐fold uptake of RGD‐VEGF‐EVs compared to naïve EVs (Figure 4b). 3D confocal imaging verifies cellular in vivo internalization of EVs (Figure 4c).

### 3.5 Effect of Bioengineered‐EVs on Atrophy Volume and Functional Recovery After Ischemic Stroke

**FIGURE 5:**
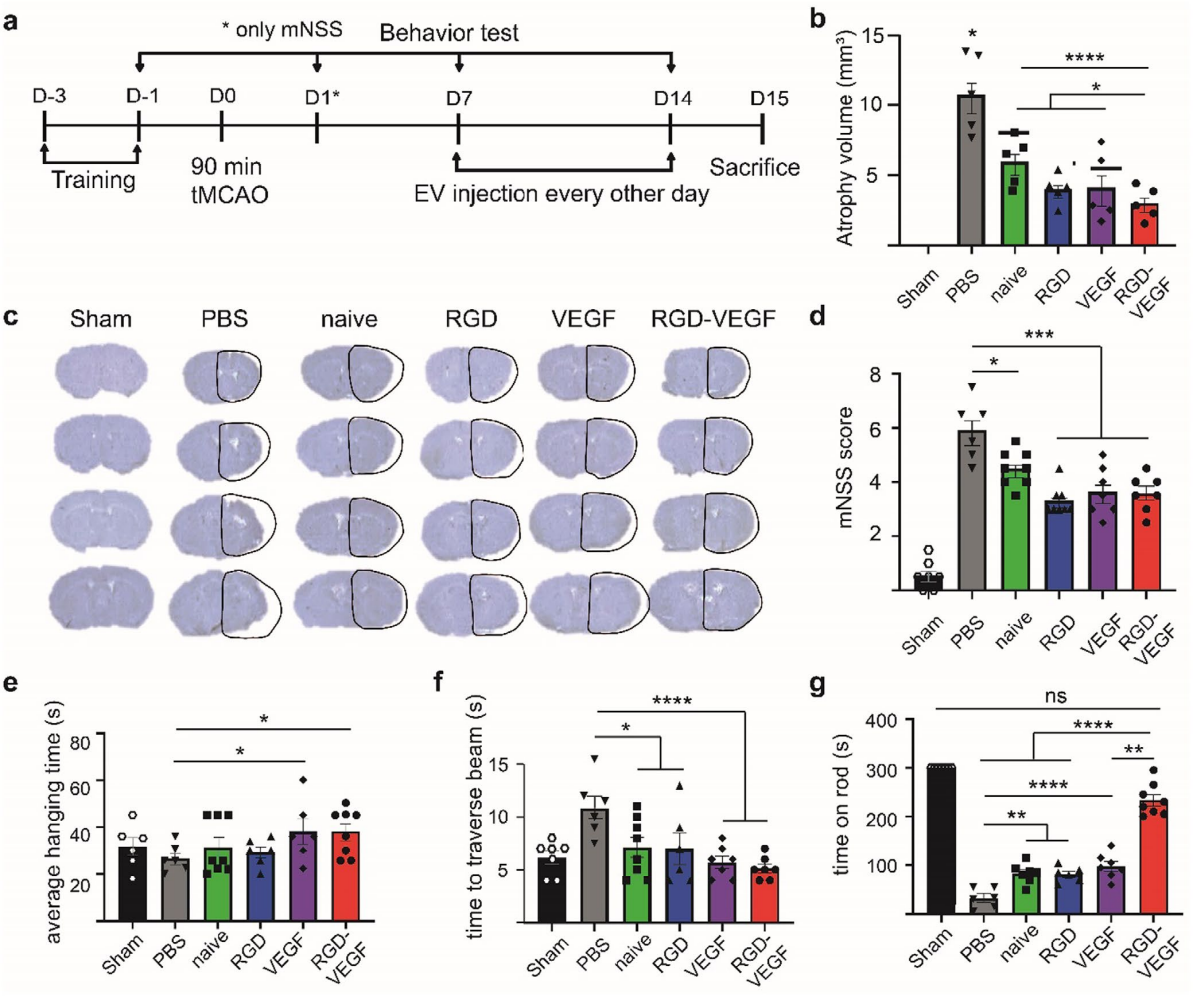
Effect of bioengineered EVs on atrophy volume and functional recovery after ischemic stroke. (a) Time line of in vivo experiment assessing the therapeutic efficiency of dual‐functionalized EVs. (b) Quantification of brain atrophy volume corresponding to (c). Atrophy volume was calculated by subtracting the volume of the ipsilateral hemisphere from that of the contralateral side and expressed as absolute volume. *Above PBS represents statistical significance to all other treatment groups. *N* = 5 per group. (c) Representative Nissl‐stained brain sections from animals treated with various bioengineered EVs. The line shows the outline of the contralateral hemisphere projected onto the infarcted hemisphere. (d) mNSS scores on D14 after tMCAO. *N* = 6–8 per group. (e) Average hanging time in the hanging wire test on D14 after tMCAO. *N* = 6–8 per group. (f) Time required to traverse the beam in the balance beam test on D14 after tMCAO. *N* = 6–8 per group. (g) Time spent on the rod in the rotarod test on D14 after tMCAO. *N* = 6–8 per group. Statistical significances: ns = not significant; *p < 0.05; **p < 0.01; ***p < 0.001; ****p < 0.0001 vs. Control.

Next, we investigated the therapeutic effect of bioengineered EVs after ischemic stroke (Figure 5a). Naïve and single‐functionalized NSC‐EVs reduce atrophy volume after ischemic stroke. Moreover, treatment with RGD‐VEGF‐EVs further reduces atrophy volume by 71.8% compared to treatment without EVs and by more than 50% compared to treatment with naïve EVs or single‐functionalized EVs (Figure 5b,c).

Furthermore, we assessed functional recovery with behavioral tests. On Day 14 after stroke, both naïve and bioengineered NSC‐EVs decrease the mNSS score (Figure 5d) and the average time needed to traverse the beam in the balance beam test (Figure 5e). In the hanging wire test, treatment with VEGF‐EVs or RGD‐VEGF‐EVs increases average hanging time (Figure 5f). RGD‐VEGF‐EVs improved time spent on the rod in the rotarod test compared to naïve or single‐functionalized EVs, up to a comparable level to healthy mice (Figure 5g).

### 3.6 Bioengineered EVs Promote Angiogenesis After Ischemic Stroke

**FIGURE 6:**
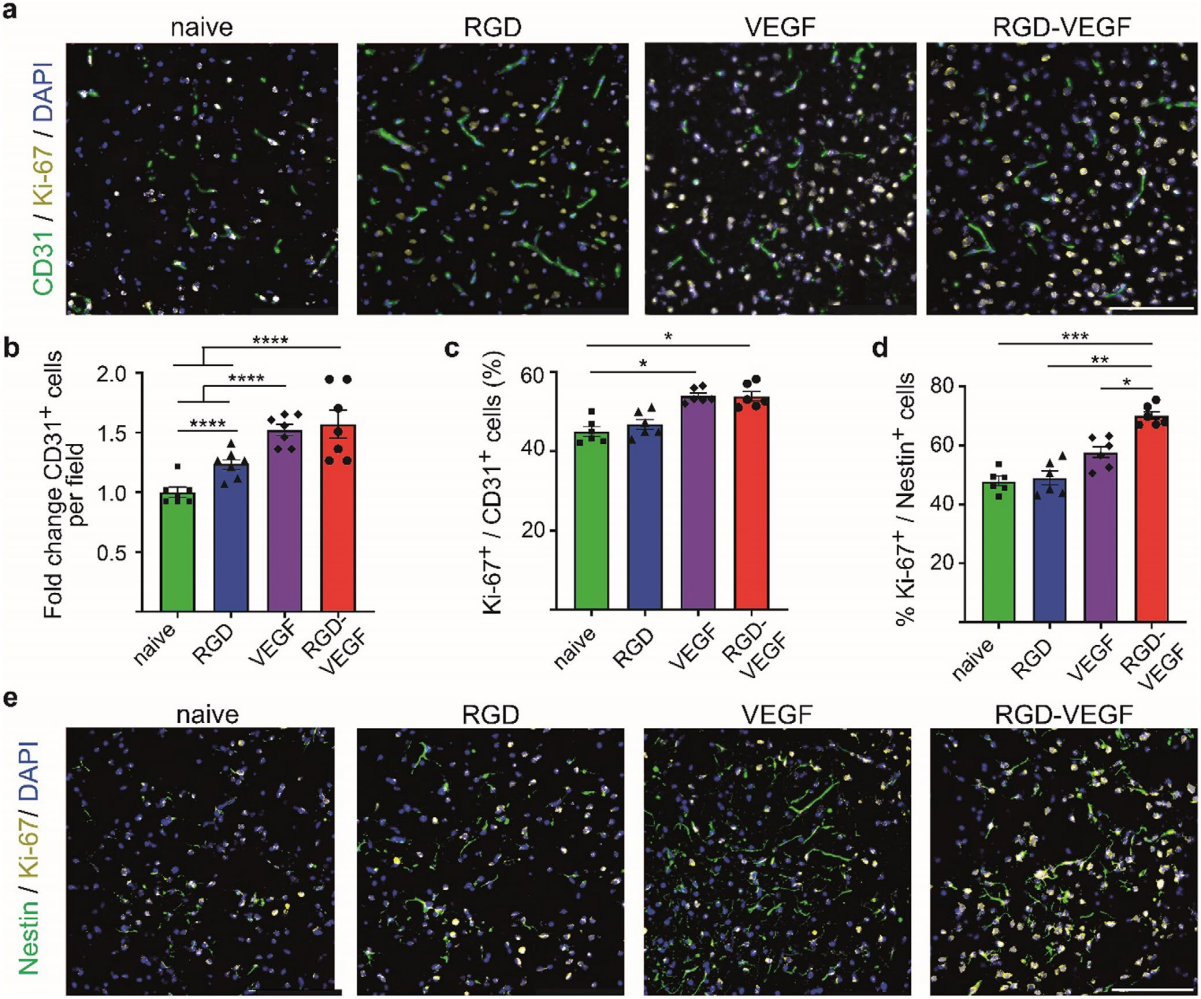
Effect of bioengineered EVs on angiogenesis and neurogenesis after ischemic stroke. (a) Representative images of proliferating endothelial cells after treatment with bioengineered EVs with (b) bar graph showing quantification of vessel density as number of vessels per field normalized to naïve EVs and (c) bar graph showing quantification of percentage of proliferating endothelial cells. *N* = 6–7 per group. (d) Bar graph showing quantification of proliferating neural progenitor and stem cells after treatment with bioengineered EVs corresponding to (e). (e) Representative images of proliferating neural progenitor and stem cells after treatment with bioengineered EVs. *N* = 6 per group. Statistical significances: *p < 0.05; **p < 0.01; ***p < 0.001; ****p < 0.0001 vs. Control.

To determine the mechanism behind the neurobehavioral improvements and reduced atrophy volume after treatment with bioengineered EVs, we investigated their effect on angiogenesis (Figure 6a). Mice treated with RGD‐VEGF‐EVs showed a 1.6‐fold increase in vessel density compared to treatment with naïve EVs (Figure 6b). Moreover, the amount of proliferating endothelial cells is increased in mice treated with RGD‐VEGF‐EVs compared to treatment with naïve EVs (Figure 6c). Furthermore, we took the first steps to see if RGD‐VEGF‐EVs have an effect on neurogenesis. We observe an increase in the proliferating neural stem and progenitor cells in mice treated with RGD‐VEGF‐EVs (Figure 6d,e).

## 4 Discussion

In the present study, we dual‐functionalized EVs with RGD surface modification for increased targetability and SLO‐mediated VEGF encapsulation for increased therapeutic effects. RGD surface modification increases EV uptake in endothelial cells by five‐fold compared to uptake of naïve EVs due to its high binding affinity to αVβ3. Moreover, dual‐functionalized EVs promote endothelial cell migration in vitro and accumulate in the infarcted brain. Therapeutic effects of dual‐functionalized EVs can be observed in a reduction in atrophy volume by 50% compared to treatment with naive or single‐functionalized EVs, improved functional outcomes with balance and motor coordination restored to near‐healthy levels, as well as signs of promoted angiogenesis and potential neurogenesis.

### 4.1 Characterization of Dual‐Functionalized EVs

We bioengineered dual‐functionalized EVs combining bio‐click chemistry and EV loading. Commonly used active loading methods show a low loading efficiency of < 30% [21, 22, 32, 33, 34]. Here, we achieve a high loading efficiency of > 70% with SLO as the active loading agent. Furthermore, to our knowledge, we are the first to demonstrate that bio‐click chemistry does not affect EV loading with SLO and vice versa. Moreover, both bioengineering steps do not affect the size or morphology of EVs. In addition, we observed a reduced drug release from RGD‐VEGF‐EVs, which may be attributed to RGD conjugation on the EV surface. Comparison with existing literature remains challenging due to the limited number of studies utilizing bio‐click chemistry for EV surface modification [17, 18, 21]. Among these, only Khan et al. report a drug release profile following conjugation with RVG29. In their study, no significant difference in drug release between modified and unmodified EVs was observed [21]. Notably, RVG29 is a relatively large peptide, consisting of 29 amino acids and a molecular weight of approximately 3300 Da [35], whereas RGD is a much smaller tripeptide (~1.0 nm) [36, 37]. Due to its size and structural simplicity, a higher number of RGD peptides may bind to DBCO on the EV surface, potentially forming a peptide‐rich layer that alters membrane properties, such as rigidity, thereby affecting drug release kinetics. A delay in drug release could reduce premature drug loss during circulation, enhancing delivery efficiency to the ischemic brain, making dual‐functionalized EVs more effective for therapeutic applications.

### 4.2 Enhanced Targeting via RGD Functionalization

The RGD‐peptide exhibits a high binding affinity to αVβ3, which is selectively upregulated on endothelial cells under ischemic conditions [15]. Our in vitro and in vivo experiments confirm that surface functionalization of NSC‐EVs with RGD significantly enhances their binding and uptake by endothelial cells through interaction with αVβ3. Furthermore, our findings indicate that the inclusion of VEGF as a therapeutic cargo within EVs contributes to enhanced targeting efficiency.

This dual‐functionalization creates a self‐reinforcing positive feedback loop, where VEGF not only promotes angiogenesis but also amplifies the expression of its own docking receptor αVβ3 [15], thereby enhancing subsequent EV uptake as well as therapeutic delivery. Such a mechanism has not been described in the context of EV‐based therapies and represents a conceptual advancement in targeted drug delivery. Compared to earlier strategies that either rely on non‐targeted EVs [38] or deliver VEGF as a free protein or gene therapy [39, 40], our approach uniquely combines spatial precision with cargo potency, resulting in improved EV accumulation at the ischemic site and greater therapeutic impact. By integrating targeting and treatment in a feedback‐enhanced system, this dual‐functionalization strategy holds potential for superior efficacy and reduced off‐target effects in stroke therapy.

In addition, we observed RGD‐mediated targeting not only to endothelial cells but also to neurons, implying a broader applicability of this targeting strategy for neurovascular repair. These combined effects significantly improve the localization and therapeutic efficacy of NSC‐EVs in ischemic brain regions [15].

### 4.3 VEGF Loading Enhances Therapeutic Potency

Our in vitro experiments confirm that VEGF‐loaded EVs significantly enhance endothelial cell migration, indicating (a) successful delivery of VEGF as well as (b) preservation of its bioactivity. In vivo, treatment with RGD‐VEGF‐EVs leads to promotion of angiogenesis, as evidenced by increased vessel density and endothelial proliferation in the peri‐infarct zone. This confirms that dual‐functionalized EVs can selectively target ischemic endothelial cells and trigger reparative vascular responses. In addition to vascular regeneration, our histological analysis shows that treatment with RGD‐VEGF‐EVs substantially reduces atrophy volume in mice 15 days after ischemia compared to animals treated with naïve or single‐functionalized EVs. Importantly, these anatomical improvements correlate with enhanced functional recovery, particularly in sensorimotor performance, underscoring the therapeutic relevance of targeted EV delivery. Strikingly, only RGD‐VEGF‐EV treatment results in increased proliferation of neural stem and progenitor cells within neurogenic niches, suggesting a neurogenic response specifically associated with dual‐functionalized EVs. This finding implies that the regenerative impact of VEGF is maximized when it is delivered in a targeted fashion to the ischemic brain.

Together, these findings underscore a synergistic relationship between targeted delivery and therapeutic cargo: While VEGF alone can promote vascular regeneration, its full neuroregenerative potential appears to be unlocked only when delivered efficiently to the injury site via RGD‐mediated targeting. Thus, dual‐functionalization of EVs represents a critical strategy to enhance both vascular and neural regeneration, ultimately leading to improved structural and functional outcomes following ischemic stroke.

### 4.4 Timing of Delivery and Biological Rationale

We initiated EV treatment 7 days following tMCAO, a timepoint chosen based on a strong biological rationale: (1) NSC proliferation peaks at 7 to 14 days post‐stroke [41], (2) angiogenesis begins within hours of stroke onset, also peaking at 7 to 14 days after stroke [15, 42], and (3) VEGF should be applied at least 48 h after stroke to avoid adverse effects like BBB leakage [43, 44, 45]. Thus, 7 days after stroke is an ideal time point to start treatment with bioengineered EVs loaded with VEGF, to capitalize on a period of peak integrin αVβ3 expression and endogenous neurogenesis without compromising safety.

### 4.5 Limitations and Future Directions

Despite our promising findings, some limitations must be acknowledged. First, we did not quantify EV yield and potential loss during the engineering process. Previous work by Ruan et al. reports up to 60% EV loss following bio‐click modification [26]. It is likely that our two‐step process incurs a similar loss. Future work needs to aim at optimizing the workflow to enhance EV recovery, which will be essential for clinical scalability and cost‐effectiveness.

Second, our evaluations focused on short‐term therapeutic effects up to 14 days post‐stroke. To fully assess the translational potential of RGD‐VEGF‐EVs, longer‐term studies are required. These may include chronic outcome assessments, long‐term safety profiling, particularly regarding immunogenicity and off‐target effects. Long‐term studies may also investigate the effect of RGD‐VEGF‐EVs on neurogenesis in more detail, including the investigation of integration and maturation of new neurons. We expect that in a long‐term study, RGD‐VEGF‐EVs would show more distinct functional recovery compared to our results assessed at 14 days after tMCAO, as angiogenic and neurogenic effects would lead to more brain repair and remodeling.

In this study, VEGF was chosen as a proof‐of‐concept therapeutic cargo. However, the modularity of our EV platform enables loading with other agents such as neurotrophic factors or small interfering RNAs. Future research could explore a combination of loaded cargo or sequential release strategies to further enhance the efficacy of EV‐based therapies.

In summary, we bioengineered dual‐functionalized EVs to enhance the targeting and therapeutic potency of NSC‐derived extracellular vesicles for the treatment of ischemic stroke. With RGD as a targeting peptide to integrin αVβ3 on activated endothelial cells and VEGF as a therapeutic drug and αVβ3‐expression enhancer, we create a positive feedback loop for the uptake and consequently therapeutic efficiency of RGD‐VEGF‐EVs. Our results highlight the importance of synergistically integrating targeting ligands and bioactive cargo in EV design. This dual‐modified platform not only improves outcomes in preclinical stroke models but also sets the stage for future translational development of EV‐based therapies in neurovascular diseases.

## Author Contributions

**Victoria Shi:** planning, execution, and analysis of experiments, drafting manuscript. **Shengju Wu**, **Qianyuan Lian**, and **Rubing Shi:** tMCAO model. **Ze Liu:** behavior tests. **Tongtong Xu** and **Shiyu Deng:** TEM and confocal image acquisition. **Xinfa Shao:** cell culture. **Anja Beckmann**, **Wanlu Li**, and **Yaohui Tang:** data interpretation and discussion. **Zhijun Zhang**, **Carola Meier**, and **Guo‐Yuan Yang:** study design, data interpretation, discussion, and manuscript revision.

## Conflicts of Interest

The authors declare no conflicts of interest.

## Data Availability

The data that support the findings of this study are available from the corresponding author upon reasonable request.
